# HydroCrowd: a citizen science snapshot to assess the spatial control of nitrogen solutes in surface waters

**DOI:** 10.1038/srep16503

**Published:** 2015-11-12

**Authors:** Lutz Breuer, Noreen Hiery, Philipp Kraft, Martin Bach, Alice H. Aubert, Hans-Georg Frede

**Affiliations:** 1 Institute for Landscape Ecology and Resources Management (ILR), Research Centre for BioSystems, Land Use and Nutrition (IFZ), Justus Liebig University Giessen, Heinrich-Buff-Ring 26, 35390 Gießen, Germany; 2 Centre for International Development and Environmental Research, Justus Liebig University Gießen, Senckenbergstraße 3, 35392 Gießen, Germany

## Abstract

We organized a crowdsourcing experiment in the form of a snapshot sampling campaign to assess the spatial distribution of nitrogen solutes, namely, nitrate, ammonium and dissolved organic nitrogen (DON), in German surface waters. In particular, we investigated (i) whether crowdsourcing is a reasonable sampling method in hydrology and (ii) what the effects of population density, soil humus content and arable land were on actual nitrogen solute concentrations and surface water quality. The statistical analyses revealed a significant correlation between nitrate and arable land (0.46), as well as soil humus content (0.37) but a weak correlation with population density (0.12). DON correlations were weak but significant with humus content (0.14) and arable land (0.13). The mean contribution of DON to total dissolved nitrogen was 22%. Samples were classified as water quality class II or above, following the European Water Framework Directive for nitrate and ammonium (53% and 82%, respectively). Crowdsourcing turned out to be a useful method to assess the spatial distribution of stream solutes, as considerable amounts of samples were collected with comparatively little effort.

In the past couple of years, *crowdsourcing* has become a more frequent practice. Crowdsourcing is a form of citizen science, involving citizens from the non-scientific community in academic research^1^. Howe^2^ defined crowdsourcing as “[…] the act of a company or institution taking a function once performed by employees and outsourcing it to an undefined (and generally large) network of people […]”. Volunteer bird surveys are the longest-running and most common field of application of citizen science. Between 2005 and 2010, more than 90 papers were published on this issue, mainly addressing ecological and methodological aspects^3^. In the context of global biodiversity mapping, crowdsourcing has great potential to expand databases of species occurrence^4^. Advances in telecommunication technology have led to a new type of citizen science. Internet applications use crowdsourcing to enlist people to monitor ecological or environmental data covering broad areas of interest. The applications often allow volunteers to access and interpret the data that they collect^2^. Specific research applications (‘apps’) for smartphones represent a powerful combination of sensors, geographical location and information transfer and they warrant better exploitation by ecological and environmental researchers^5^. The crowdsourcing tool Geo-Wiki was developed to increase the amount of *in-situ* land-cover data available for training, calibration and validation of global land-cover maps derived from remote sensing^6^. Since 2012, more than 500 users have added local soil data to the soil databank in the United Kingdom via the mobile phone app *mySoil*, which is termed a “democratization of soil data” by the developers of the app^7^.

Crowdsourcing has so far rarely been used in hydrology^8^, although the drift-bottle studies of the 1960 s and 1970 s^9^,^10^,^11^ can be seen as an early prototype. Thousands of bottles with paper instructions inside were released in large streams in order to understand surface currents. The finders of the bottles were asked to return the paper instructions, indicating where the bottle was found. Others have used crowdsourcing to determine water levels^12^. They equipped streams near hiking trails with measuring points, and hikers were requested to send a text message of the water level. The software package *Social.Water* was set up as a crowdsourcing tool for the acquisition of environmental data, especially hydrological measurements^13^. In the Liguria region (Italy), citizens reported a flash flood that affected them in 2011^14^. Generally, such projects are based on the voluntary involvement of a large number of citizens.

In the present study, a crowdsourcing campaign called *HydroCrowd* was used. We aimed to collect water samples throughout Germany on one specific day to investigate the spatial distribution of nitrogen. Snapshot sampling, as performed during *HydroCrowd*, provides a look at ecosystem conditions (in our case of water quality) at a specific time and under common boundary conditions^15^. Water samples were analysed for NO_3_^−^-N (nitrate-nitrogen), NH_4_^+^-N (ammonium-nitrogen) and DON (dissolved organic nitrogen). Ammonium and nitrate, mainly originating from agriculture, sewage-treatment plants and other anthropogenic driven diffuse and point sources, significantly contribute to eutrophication^16^,^17^,^18^. In 2011, the estimated nitrogen surplus in German agricultural areas was 80 kg N ha^−1^^19^. Ammonium and nitrate concentrations are two of the main determinants of the classification of water quality according to the EU Water Framework Directive (WFD, guideline 2000/60/EC). There is only a weak understanding of the impact of DON on total N in ecosystems; until recently, DON was considered to be of little importance in the N-cycle^20^, but several studies have now indicated that DON may have a significant influence on ecosystems. Perakis and Hedin^21^ showed that the contribution of DON to total N solute concentrations is much higher in unpolluted areas. However, despite generally higher concentrations of dissolved inorganic nitrogen (DIN) in the surface waters of an intensively farmed landscape in Denmark, DON also significantly contributed to the total N balance^22^. A meta-analysis also found a significant correlation between arable land and DON level^23^. A relationship between humus content and DON was shown by others^24^,^25^.

Given this background, the primary objective of the present study was to address the following question: is crowdsourcing a reasonable method for assessing the spatial distribution of nitrogen components in surface waters on a large scale? Using samples obtained as a spin-off experiment, we investigated how catchment characteristics affect the water quality in Germany in terms of nitrate, ammonium and dissolved organic nitrogen.

## Results

### Catchment characteristics

On 3 October 2013, 280 stream samples were taken in many regions of Germany, excluding eastern Germany, for which no students provided samples (see Methods). Most of the samples were taken in the Federal State of Hesse. Of 570 distributed flasks, 280 flasks were returned (a return rate of almost 50%). Delineated catchment size varied between a minimum of 1,600 m^2^ and a maximum of 28,000 km^2^ with a mean of 624 km^2^ (Table 1). 28% of all samples were taken in rivers wider than 5 m, including large rivers such as the Rhine, Main, Weser and Ems. 56% of the sampled watercourses had a stream width between 0.5 m and 5 m, and the remaining 16% were taken in streams narrower than 0.5 m. The dominating land use was forests (37%), arable land (31%) and grassland (25%). 46% of the catchments had no arable land use, 50% had no grassland and 40% had no forests. The humus content ranged between 2.5% and 22.5%, with a mean of 5.2%. The population density varied between a minimum of 28 inhabitants km^−2^ and a maximum of 2,740 inhabitants km^−2^, with a mean of 365 inhabitants km^−2^. 23% of the catchments had a lower population density than 100 inhabitants km^−2^, and 13% had a higher density than 800 inhabitants km^−2^. Seven samples identified as outliers were discarded, mainly due to sampling in streams not following the instructed conditions (for example, in town or too close to a sewage plant).

### Nitrate, ammonium and DON concentration

**Figure 1:**
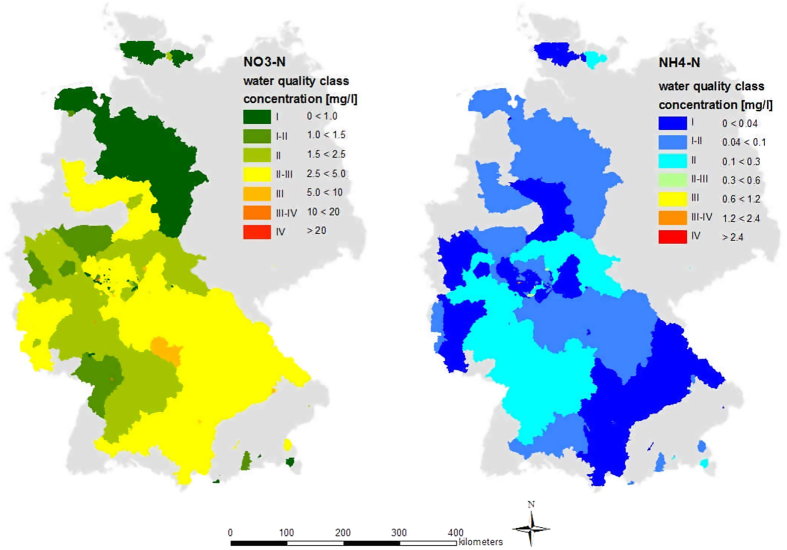
Nitrate and ammonium concentrations and respective water quality classes^27^ in the sampled catchments in Germany. Class I: no anthropogenic pollution, Class I-II: very low pollution, Class II: moderate pollution, Class II-III: significant pollution, Class III: increased pollution, Class III-IV: high pollution, Class IV: very high pollution. Maps were generated with ArcGIS 10.0, ESRI.

**Figure 2:**
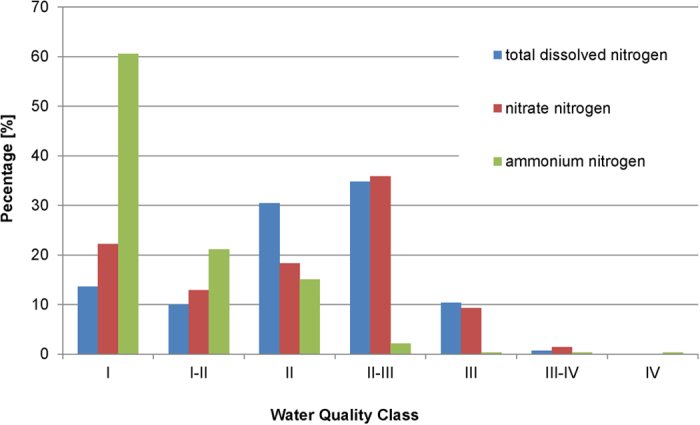
Frequency distribution [%] of samples per water quality class (for water quality class definitions, see Methods).

NO_3_^−^-N concentrations ranged from below the detection limit to above 10 mg l^−1^ (mean 2.70 mg l^−1^ ± 2.09 (one standard deviation, SD)). The results can also be accessed online^26^. In southern Germany, NO_3_^−^-N concentrations were generally slightly higher (Fig. 1) than in the north. 53% of the stream concentrations were of water quality class II or better (Fig. 2). 36% had concentrations between 2.5 mg l^−1^ and 5 mg l^−1^ (class II-III), and 11% showed concentrations of water quality class III and III-IV. There were no streams of water quality class IV. NH_4_^+^-N concentrations ranged from below the detection limit (0.07 mg l^−1^) to above 2 mg l^−1^. More than 95% of the samples were of water quality class II or above in terms of ammonium (Fig. 2).

**Figure 3:**
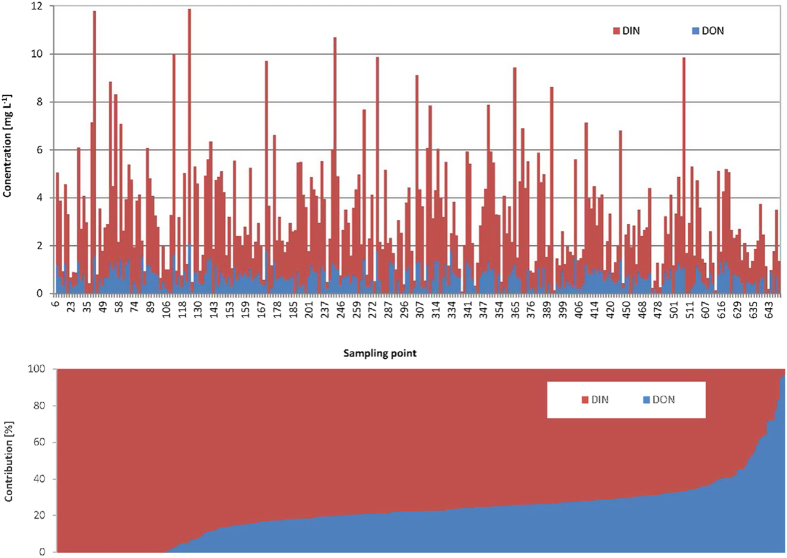
(top) Absolute concentrations of DIN and DON [mg l^−1^] per sample point; (bottom) contribution [%] of DIN and DON across the stream cohort of the *HydroCrowd* experiment.

Total Dissolved Nitrogen (TDN) concentrations, composed both of organic and inorganic nitrogen, ranged from 0.1 mg l^−1^ to almost 12 mg l^−1^ (mean 3.25 mg l^−1^ ± 2.24). DON values ranged from 0 mg l^−1^ to 2 mg l^−1^ (mean 0.64 mg l^−1^ ± 0.46) (Fig. 3). 15% of the samples contained no DON, 64% had concentrations lower than 1 mg l^−1^, and 21% showed concentrations between 1 mg l^−1^ and 2 mg l^−1^. DIN ranged from 0.01 mg l^−1^ to more than 10 mg l^−1^ (mean 2.62 mg l^−1^ ± 1.98). Given the average low NH_4_^+^-N concentration, NH_4_^+^-N can be neglected, and thus DIN was nearly equivalent to NO_3_^−^-N. The average ratio of DIN/DON was 4.8. The mean contribution of DON to TDN was 22% ± 16 (Fig. 3). More than 90% of the samples had a higher DIN concentration than DON.

### Effect of catchment characteristics on nitrate and DON concentrations

Catchment characteristics moderately affected nitrogen concentrations. NO_3_^−^-N had an average positive correlation with arable land coverage (r = 0.46) and the average humus content of the catchment (r = 0.37). A minor but significant correlation was found with population density (Table 2). However, the corrected R^2^ (18%) indicates an overall low explanatory value of the model. The DON correlation with catchment characteristics was even weaker than for NO_3_^−^-N (Table 3), and the scatter plot of the residuals obtained from the multivariate regression analysis showed heteroscedasticity.

### No effect of stream morphology (width)

The mean NO_3_^−^-N concentration of water bodies with a width >5 m was 2.4 mg l^−1^ ± 1.4 SD. The mean NO_3_^−^-N concentration of water bodies with a width 0.5 m to 5 m was 3 mg l^−1^ ± 2.4 SD, similar to water bodies smaller than 0.5 m (3 mg l^−1^ ± 3.7 SD). For DON, the ranking of water bodies was similar: width >5 m (mean 0.5 mg l^−1^ ± 0.4 SD), 0.5 m to 5 m (0.7 mg l^−1^ ± 0.6 SD), and <0.5 m (mean 0.6 mg l^−1^ ± 0.4 SD). However, we did not find significant differences between stream width classes and their mean nitrogen concentrations.

## Discussion

### Crowdsourcing for assessing the spatial distribution of nitrogen in surface waters

This snapshot provides a large view of the relevant N solutes in stream waters. Thus, in the perspective of spatial hydrological investigations, crowdsourcing is relevant. Moreover, the often limited budgets of universities prevent them from such large sampling campaigns, and crowdsourcing is a relatively low-cost, convenient method to implement.

The *HydroCrowd* project would have been impossible without crowdsourcing. Thus far, crowdsourcing has seldom been used in hydrology, and therefore we cannot compare the return rate of flasks (~50%) to those of other projects. In our opinion, it was quite a good rate of return considering the short preparation and notice time (owing to the university calendar, students were on semester leave for the three months preceding the sampling event). By its very nature, crowdsourcing success is uncertain; the numbers of returned samples and the areas from where they come are unpredictable. Therefore, we had to address the spatial distribution and representativeness of sampling as it was, even though it was not ideal, making the assumption of independence. Certain dates can be favoured to enhance widespread involvement.

A potential limitation of data quality is related to the uncertainty of the data collected by the crowd. Sample location can be inaccurately recorded and samples inadequately stored. To overcome the problem of incorrect sampling location, we crosschecked all coordinates and names of streams, nearest settlement and closest street. In cases of doubt, we contacted the person who collected the sample. How to sample and store water samples was explained during lectures to crowd participants. As the crowd consisted mainly of students focusing on environmental management, we are positive that samples were taken and stored accordingly. Finally, we only accepted water samples returned to the institute cold or, even better, frozen.

Overall, we see crowdsourcing as a suitable method, although for a territory the size of Germany, the quantity of samples was a bit too low in our experiment. For future studies, it would be better to choose smaller areas, such as federal states or administrative districts, and increase the size of the targeted crowd and communication about the event. An option could be to team up with kindergartens or schools. Involving associations for ecological conservation, fishing clubs and water sport clubs could ensure a wider spatial distribution and more samples. However, all of this would dramatically increase the workload of distributing sampling material and collecting water samples. Thus, the design of the target group is a trade-off between cost, time and research objectives.

### Water quality regarding N-compounds and the effect of catchment characteristics

The EU WFD requires all surface waters in Europe to be in water quality class II or higher by 2015. According to our snapshot, 53% of the sampled water bodies are in class II or above in terms of nitrate (Fig. 2). The water quality was improved in comparison to other studies. In 2004, only 13% of the water bodies were classified as water class II or better^27^. We observed a related decrease of samples ranked as water class III from 38% in 2004 to 9% in our study. The ammonium concentrations reveal the same upward trend as nitrate. In 2004, two-thirds of the samples were in water quality class II^27^, whereas we detected 82% in category II or above. However, we note that the results of our study are based on a single snapshot, and replications at different seasons are required to confirm our findings. A further methodological problem occurs in comparing such reported data with those obtained from crowdsourcing. Large numbers of samples collected by state agencies are not taken on the same day. Thus, a direct comparison of these data with our crowdsourcing data is potentially influenced by differences in meteorological and hydrological boundary conditions.

It appears that nitrate concentrations were lower in northern Germany than in the south (Fig. 1). However, this perception is mainly based on a single sample of the large Weser catchment in the north, displaying water quality class I. According to our statistical analyses, arable land has the strongest influence (beta = 0.34) on NO_3_^−^-N concentration, which is consistent with other studies^28^,^29^. This is the result of diffuse N inputs to streams via N-fertilizer application. Even a decrease in the fertilizer amount would not directly improve this situation, as soils operate as N sinks and N sources for years^30^. Humus content had the second strongest influence on NO_3_^−^-N. Soils with high humus content can mineralize a substantial amount of nitrate, which is susceptible to leaching^31^. The population density had nearly no influence (beta = −0.05) on N-solute concentrations. This was unexpected, as settlements (and thus population density) are associated with sewage treatment plants, which increase anthropogenic pollution by point source inputs^32^. It is possible that a relatively high background concentration of nitrate caused this result; there are almost no regions in Germany without settlements, and it is possible that fertilizer application leads to high nitrate concentrations, which would suggest that the population density has no bearing on nitrate concentrations.

Overall, our regression model explains only a little of the observed variance, with R^2^ = 0.18 (Table 3), whereas others found that agricultural cover had a high influence (R^2^ = 0.69) on nitrate concentrations^28^. In a Danish agricultural dominated landscape, soil type, texture, and to a lesser extent, agricultural land use explained 98% of the variance^22^. Therefore, we conclude that, in our case, there are additional drivers of nitrate concentration that were not part of our study. Precipitation could be such a trigger, mobilizing soil N and subsequent nitrate leaching^31^. In a paired catchment experiment with similar land cover, soil types and mineralization rates, nitrate concentrations differed by a factor of ten, which was attributed to differences in the slopes that control water table dynamics^33^. While this is a potential explanation for smaller scale catchments, we doubt that this will explain any differences on a larger scale.

### Effect of catchment characteristics on DON

Perakis and Hedin^21^ showed that DON is the dominant N form in unpolluted areas, with an average of 80% of total N (NO_3_^−^ -N represented only 5%). Based on this, van Breemen^20^ postulated that there is a shift from dissolved organic nitrogen to inorganic nitrogen stream concentrations and loads along a gradient from nutrient-poor to nutrient-rich landscapes. Accordingly, DON should be less important in closing the N balance of intensively farmed landscapes. However, despite the lower contribution of DON than DIN to the total N balance, DON should not be ignored^22^,^34^. This is also supported by our observations in the *HydroCrowd* experiment, where we found an average contribution of DON of approximately 22%.

The question is whether and how human activities and general catchment characteristics influence DON concentrations. Our statistical analyses reveal only weak correlations of DON with both humus content and arable land. No significant correlation was found with population density. The resulting regression analyses were very weak and distorted by heteroscedasticity. Because humus is the most important dissolved organic matter source in soil^35^, we expected a stronger relation of humus content and DON, as found by others^24^,^25^.

Vegetation cover can be an important source of DON in soils^36^. Thus, we expected a correlation between vegetation cover and DON, which is demonstrated by the relationship between land use and DON found elsewhere^23^. Furthermore, there is increasing evidence that specific plants can take up significant levels of organic nitrogen for their metabolism^37^,^38^,^39^, strengthening the likelihood of a correlation of DON and land use. However, such a correlation was weak.

Referring again to the results of Perakis and Hedin^21^, our DON/DIN ratio showed an inverse relationship. In more than 90% of our samples, DIN concentrations were higher than the DON concentrations. Previous studies also showed that DIN is significantly higher than DON in areas with anthropogenic pollution^22^,^40^. Considering catchments for which DON contributes more than 70% to the TDN, it appears that in almost three-quarters of cases, forest is the predominant land use type. This is consistent with previous studies, which demonstrated a higher DON fraction in forest catchments than in more human-dominated areas, such as arable land^39^,^41^.

According to Pellerin *et al.*^42^, DIN concentrations increase with the percentage of developed land, whereas DON concentrations remain constant. Such a general threshold could also be depicted for our cohort of *HydroCrowd* streams. Despite this general observation, there is a need to better understand the composition of DON and to gain further insights into potential relations between anthropogenic N inputs, natural drivers and instream DON concentrations. We would also like to add that our DON estimates should be considered carefully. DIN:TDN ratios of >0.6 can lead to uncertain estimates if DON is calculated as the difference of TDN minus DIN, as is the case in our study^43^. Therefore, future assessments of DON should also consider more sophisticated analytical procedures such as size excluding chromatography, rather than simple difference-based assessments. To investigate the different N sources in the landscape, stable nitrogen isotopes provide a powerful tool in the partitioning of different inputs^40^,^44^,^45^. We conclude that there are a number of open questions for which highly spatially distributed sampling campaigns could provide further insights into nitrogen pools and processes on a large scale – a topic that could be supported by citizen science projects.

## Conclusions

Crowdsourcing is a useful method with regard to the assessment of the spatial distribution of substances in the aquatic environment^8^. A large amount of data can be collected with relatively little effort. Our own experience with the students of the Justus Liebig University Giessen as a crowdsourcing collective confirmed that collecting data for use in a topical research project is highly motivating and provides opportunities for interacting with students^1^.

Further studies on crowdsourcing in hydro-biogeochemistry should address a thorough quality check of its sampling strategy. A full investigation of the trade-off of “less, but more accurate data obtained by trained technicians or scientists” versus “more, but potentially less accurate data taken by a HydroCrowd” is needed. Repeating the experiment, investigating the motivation of participants, studying the sources of sampling errors (including sample storage by the crowd) and accuracy of reported sampling location is needed to quantify the method’s overall data uncertainty.

Apart from the general results related to water quality classes and their trends in Germany, this study also indicates the importance of DON to the total dissolved nitrogen concentration. Despite the generally accepted assumption that DON dominates N solutes in remote places of the world^21^, we show that even under mid-European conditions, DON accounts for approximately 22% of the total dissolved N. We see this as an important aspect of future water quality studies and investigations of landscape-scale N cycles.

## Methods

### Crowd identification and crowdsourcing area

First, to advertise about the snapshot-sampling event, a group of citizens was targeted. Students of the faculty of Agricultural Science and Environment Management at Justus Liebig University Giessen (JLU) were informed about this experiment during lectures and by email. This student crowd was adequate due to their easy availability in addition to their interest in hydrology and biogeochemistry. Additional participants were attracted through word-of-mouth recommendation. Informed consent to use samples for further analyses and publish results was obtained from all participants. A Facebook page and website were set up for further advertisement and up-to-date information. Given that the crowd was mainly composed of undergraduate students of JLU, we assumed that most of the samples would be taken within Germany, with a regional focus on the Federal State of Hesse. When sampling, the participants had to fill out a questionnaire.

### Crowd sampling and sample storage

The sampling took place on 3 October 2013 (German Unity Day, a bank holiday). This was advantageous for a wider distribution of sampling, as many students returned to their home town. Crowdsourcing participants were asked to return flasks immediately after sampling, between 7^th^ and 18^th^ October. As the start of the next semester was in mid-October, students returned to the university, facilitating an efficient sample collection at the institute. The requirements were to take samples only from flowing waters and not from lakes or the ocean, not under channels or inside towns and to avoid those in proximity to sewage-plants. Every participant received a 100-ml PE-sampling-flask cleaned by ultra-pure water and the following instructions: “rinse flasks with stream water prior sampling, fill out the questionnaire, store samples in a cold place or freeze them” and finally “return the samples in a cooled storage container by post or personally to our institute at JLU Giessen before 18 October 2013, at the latest”. To prevent microbial nitrogen turnover and degradation, the participants were asked to keep samples frozen or at least to store them at a cool temperature until delivery to the laboratory, where samples were stored frozen until the analyses started. The questionnaire included questions about sampling location (coordinates obtained by GPS/smartphone or digital maps, stream name, name of next settlement and street), stream width class (<0.5, 0.5 to 5, and >5 m), colour and turbidity of stream (qualitative information), precipitation antecedent sampling (on that day, a day before, two days before), air temperature (<5, 5−10, 10–15, 15–20, and 20–25 °C), name of participant and email address.

### Laboratory analyses

Before the analyses, samples were thawed at room temperature. All water samples were filtered through 0.45 μm polypropylene syringe filters. The total dissolved nitrogen (TDN) was determined by catalytic high temperature oxidation and infrared spectrometry (liquiTOC, Elementar, Hanau, Germany), after acidification with 37% hydrochloric acid. NH_4_^+^-N and NO_3_^−^-N were analysed by continuous flow analysis photometry (AutoAnalyzer3, SEAL Analytical, Norderstedt, Germany). NH_4_^+^-N was determined according to the standard method DIN 38406 (ISO/DIS 11732, detection limit 0.07 mg l^−1^). NO_3_^−^-N was determined according to the standard method DIN 38405 (ISO/DIS 13395, detection limit 0.006 mg l^−1^). DIN was assessed as composed of NO_3_^−^-N and NH_4_^+^-N, neglecting potential but low contributions of NO_2_^−^-N. DON was calculated by subtracting DIN from TDN.

### Data analyses

**Figure 4:**
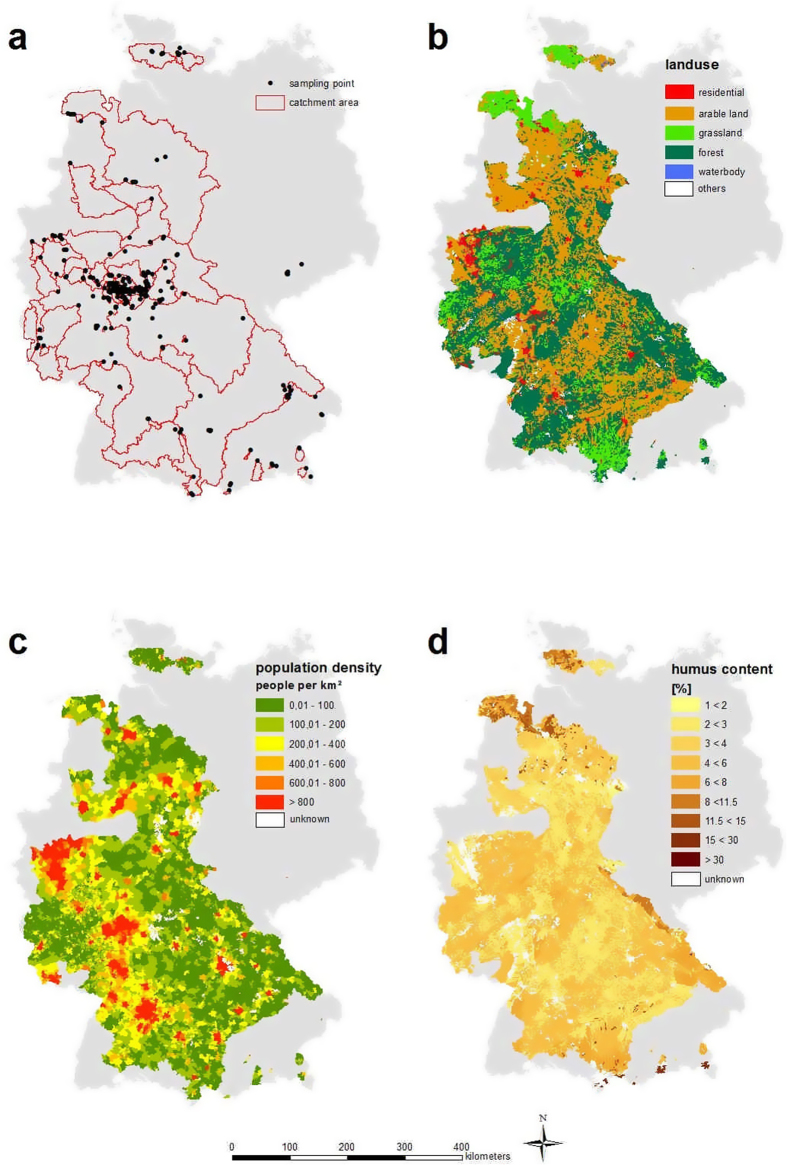
Crowdsourcing area: (**a**) study area with sampling points and catchment area; (**b**) main land use classes^49^; (**c**) population density^52^; (**d**) topsoil humus content^48^; territory of Germany in grey. Maps were generated with ArcGIS 10.0, ESRI.

Questionnaire data were transferred into a spreadsheet application (Excel 2007, Microsoft, Washington, USA). NO_3_^−^-N, NH_4_^+^-N and TDN concentrations were converted into seven classes^27^ used to evaluate water quality. Spatial analyses and maps (Fig. 4) were performed using a geographic information system (ArcGIS 10.0, ESRI, California, USA). The geographic coordinates of the samples were imported to ArcGIS. Using Spatial Analyst and a 50-m digital elevation model from Germany^46^, catchment delineation, flow direction and flow accumulation from the catchment area were derived for each sampled point. Soil types and humus content were recorded from digital maps^47^,^48^. The main land use classes were obtained from CORINE Land Cover^49^ and further grouped into six categories by means of an identification key^50^. Population density was calculated for each administrative unit of municipalities^51^ from population data^52^. The average values of humus content and population density of a catchment were calculated as area-weighted means. Catchment areas with unknown humus content were assigned the humus content of their nearest neighbour catchment.

For statistical calculations, we used SPSS 15.0 (IBM Corporation, California, USA). We visually inspected the dataset for outliers (boxplots) and performed an outlier analysis. Seven outliers were deleted for further statistical analyses. We checked why outliers occurred and were able to identify incorrect sample collection (e.g., flask was only halfway filled) and sampling location (e.g., directly behind sewage treatment work or within a settlement). Data were then tested for normal distribution using a Kolmogorov-Smirnov test and QQ plots. Data were normally distributed. Nevertheless, we applied both parametric and non-parametric statistical methods. Both provided similar results. We used a 2-tailed Spearman-Rho correlation analysis to detect correlations between N solutes and landscape characteristics. To test the significance of differences between mean stream N concentrations and classes of stream width, we used a Kruskal-Wallis test. Only five samples were taken in areas where rainfall occurred within two days prior to the sampling date; thus, statistical analysis of the effect of precipitation antecedent to the date of sampling could not be performed. Finally, we applied multivariate regression analysis to set up an empirical model for explaining stream N solutes by catchment characteristics. We checked for multicollinearity using the *tolerance* and the *variance inflation factor* in SPSS. Both indicated no multicollinearity in our regression models. For the evaluation of heteroscedasticity, we visually inspected histograms, PP plots and scatter plots of the residuals.

Independence of data is an assumption for some of the statistical analyses mentioned above. As we did not pre-define sample locations for the crowd, some samples were taken in close vicinity to each other or within the same stream but some distance downstream. Both cases question the independence assumption. However, after a thorough check of our dataset, (i) only a few cases of adjacent samples occurred, and (ii) water bodies in which samples were taken at several locations along the reach showed a change in land use (i.e., settlements in between sampling location or a change from forest (upstream) to agricultural land used (downstream)). Overall, as the majority of samples were independent, we decided to treat the entire cohort of samples as independent.

## Additional Information

**How to cite this article**: Breuer, L. *et al.* HydroCrowd: a citizen science snapshot to assess the spatial control of nitrogen solutes in surface waters. *Sci. Rep.* **5**, 16503; doi: 10.1038/srep16503 (2015).

## Figures and Tables

**Table 1:** Frequency distribution of catchment characteristics in the sampled catchments.

**Catchment area [km^2^]**	**<1**	**1–100**	**100–300**	**300–1,000**	**>1,000**
Percentage [%]	50	35	4	4	7
**Population density /km^2^**	<100	100–200	200–400	400–800	>800
Percentage [%]	23	34	23	7	13
**Topsoil humus content [%]**	2–3	3–4	4–6	6–8	>8
Percentage [%]	20	8	55	10	6
**Part of land use [%]**	0	0–20	20–60	60–80	>80
Percentage arable land [%]	46	14	13	7	20
Percentage forest [%]	37	11	23	8	21
Percentage grassland [%]	50	18	13	3	16

**Table 2:** Spearman’s correlation summary at a 5% level (n = 273).

		NO_3_^−^-N	DON
Arable land	correlation coefficient	0.456^**^	0.134^*^
	Sig. (2-tailed)	0.000	0.027
Humus content	correlation coefficient	0.374^**^	0.142^*^
	Sig. (2-tailed)	0.000	0.019
Population density	correlation coefficient	0.123^*^	0.012^n.s^
	Sig. (2-tailed)	0.042	0.838

Level of significance: **p < 0.01; *p < 0.05; n.s. = not significant.

**Table 3:** Corrected R^2^ and standardized beta coefficients of multivariate regression analyses.

	NO_3_^−^-N	DON#
R^2^ corrected	0.175	0.047
Arable land	0.335^*^	0.03^n.s.^
Humus content	−0.159^*^	−0.216^*^
Population density	−0.054^n.s.^	−0.115^n.s.^

^#^Scatter plot of residuals indicated heteroscedasticity, *significant, n.s. = not significant.
